# Abnormal Brain Protein Abundance and Cross-tissue mRNA Expression in Amyotrophic Lateral Sclerosis

**DOI:** 10.1007/s12035-023-03587-2

**Published:** 2023-08-28

**Authors:** Yanni Ma, Tingting Jia, Fengqin Qin, Yongji He, Feng Han, Chengcheng Zhang

**Affiliations:** 1 Mental Health Center and Psychiatric Laboratory, The State Key Laboratory of Biotherapy, West China Hospital of Sichuan University, Chengdu, 610041 Sichuan China; 2 Department of Neurology, The 3Rd Affiliated Hospital of Chengdu Medical College, Chengdu, Sichuan China; 3 Clinical Trial Center, National Medical Products Administration Key Laboratory for Clinical Research and Evaluation of Innovative Drugs, West China Hospital Sichuan University, Chengdu, People’s Republic of China; 4 Department of Emergency Medicine, Hainan General Hospital, Hainan Affiliated Hospital of Hainan Medical University, Haikou, China

**Keywords:** Proteome-wide association study, Protein quantitative trait loci (pQTL), Differential expression analysis, Amyotrophic lateral sclerosis (ALS)

## Abstract

Due to the limitations of the present risk genes in understanding the etiology of amyotrophic lateral sclerosis (ALS), it is necessary to find additional causative genes utilizing novel approaches. In this study, we conducted a two-stage proteome-wide association study (PWAS) using ALS genome-wide association study (GWAS) data (*N* = 152,268) and two distinct human brain protein quantitative trait loci (pQTL) datasets (ROSMAP *N* = 376 and Banner *N* = 152) to identify ALS risk genes and prioritized candidate genes with Mendelian randomization (MR) and Bayesian colocalization analysis. Next, we verified the aberrant expression of risk genes in multiple tissues, including lower motor neurons, skeletal muscle, and whole blood. Six ALS risk genes (*SCFD1*, *SARM1*, *TMEM175*, *BCS1L*, *WIPI2*, and *DHRS11*) were found during the PWAS discovery phase, and *SARM1* and *BCS1L* were confirmed during the validation phase. The following MR (*p* = 2.10 × 10^−7^) and Bayesian colocalization analysis (ROSMAP PP4 = 0.999, Banner PP4 = 0.999) confirmed the causal association between *SARM1* and ALS. Further differential expression analysis revealed that *SARM1* was markedly downregulated in lower motor neurons (*p* = 7.64 × 10^−3^), skeletal muscle (*p* = 9.34 × 10^−3^), and whole blood (*p* = 1.94 × 10^−3^). Our findings identified some promising protein candidates for future investigation as therapeutic targets. The dysregulation of *SARM1* in multiple tissues provides a new way to explain ALS pathology.

## Introduction

Amyotrophic lateral sclerosis (ALS) is a common neurodegenerative disease that damages upper and lower motor neurons, eventually resulting in muscular weakness and paralysis [1]. The pathophysiology of ALS has been shown to be significantly influenced by genetic factors, and the lifetime risk in the general population has a heritability of 52.3% [2, 3]. To study the pathogenic mechanisms underlying the disease, researchers have identified many disease-associated genes, such as *SOD1*, *TDP-43*, *FUS*, and *C9ORF72*, but there are still some ALS patients whose etiology cannot be explained [4]. Among the current drug treatments, riluzole has long been the only drug to extend the survival of patients with ALS [5]. Moreover, other treatments, such as gene therapies, are still under investigation [5]. However, the present effective therapeutic targets and strategies for ALS are still limited. Therefore, new risk genes remain to be discovered, which will also facilitate the development of new targets for disease treatment.

Previous studies have used genome-wide association studies (GWAS) to identify disease-related genetic variants in case–control studies and have achieved great success in mapping susceptibility genes for ALS [1]. However, GWAS examining genetic variation in isolation to explain the pathogenic causes of ALS is limited, and complex biological processes have been shown to be involved in the pathogenesis of ALS, such as malfunctioning proteins [6, 7]. Previous studies have highlighted the pathology of brain proteins in the development of ALS, such as *TDP-43* [8] and *FUS* [9]. These proteins play crucial roles in regulating RNA metabolism and protein homeostasis, and mutations or misregulation of their activity can lead to neurodegeneration and disease pathology [10]. Therefore, studying the abnormalities in protein abundance in the brain is essential to reveal ALS pathogenesis and to identify reliable biomarkers.

To study genetic causes and etiopathogenesis, researchers have also concentrated on other ALS-related tissues in addition to motor neurons, including skeletal muscle [11] and peripheral blood [12]. Skeletal muscle is one of the main target tissues affected by ALS [13], and studying genetic abnormalities in skeletal muscle biopsies can be correlated with clinical features and disease progression in ALS patients. In addition, changes observed in gene expression patterns in whole blood may relate to the same changes occurring within the brain [14], which may offer insight into the pathogenic mechanisms of motor neuron degeneration in ALS. Therefore, we tried to combine the strengths across tissues and utilize differential expression strategies to verify whether the high-confidence risk genes are consistently dysregulated in multiple ALS-related tissues.

**Fig. 1:**
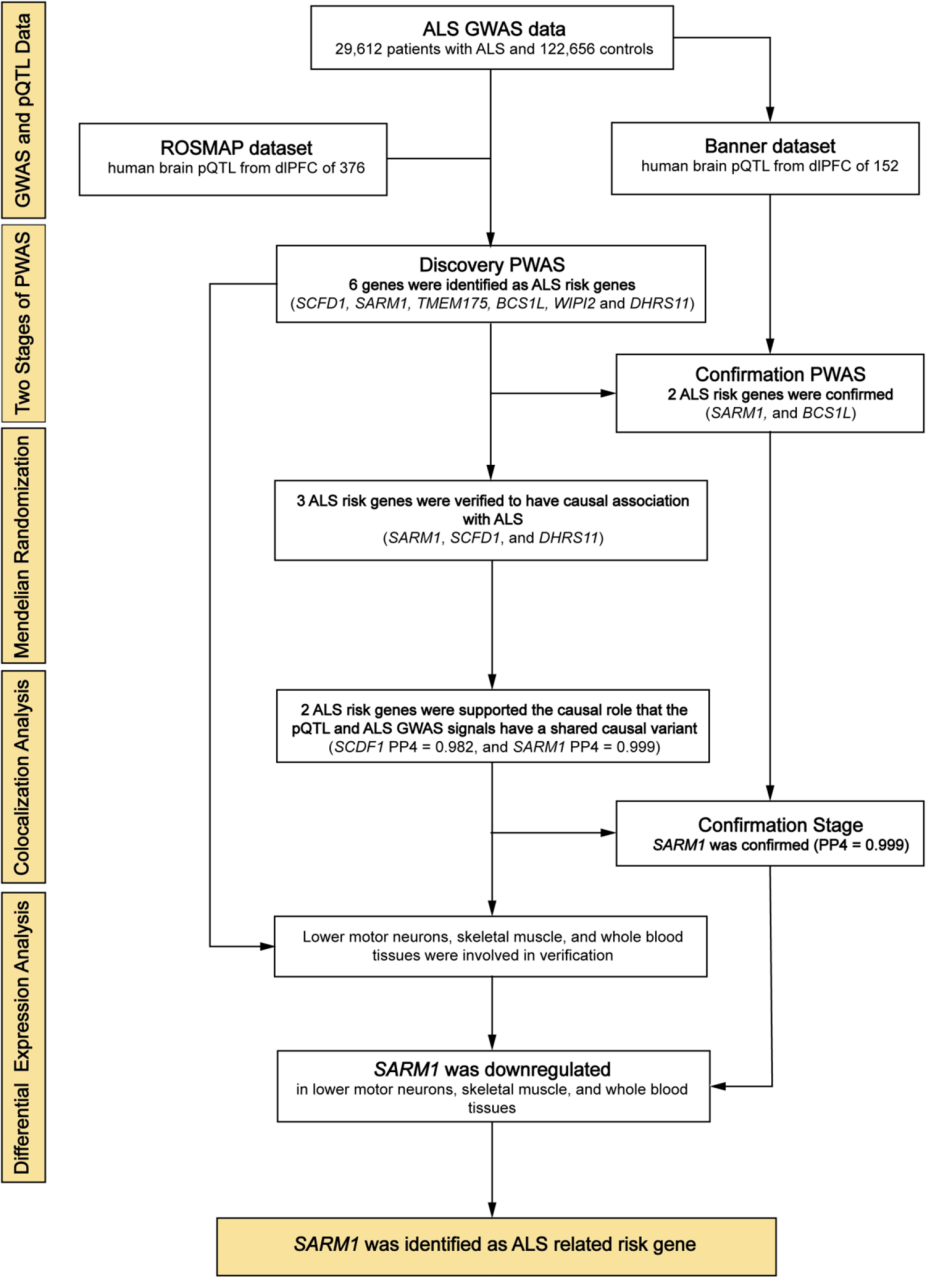
We took a four-step approach to link the proteome and ALS. First, we integrated the large ALS GWAS dataset and two independent human brain pQTL datasets (ROSMAP dataset and Banner dataset) to conduct a two-stage PWAS. Next, we applied Mendelian randomization and Bayesian colocalization analysis to validate the causal association between *SARM1* and ALS via their cis-regulated brain protein abundance. Finally, we verified the dysregulation of susceptibility genes found in the PWAS stage using differential expression analysis in lower motor neurons, skeletal muscle, and whole blood. ALS, amyotrophic lateral sclerosis; GWAS, genome-wide association study; pQTL, protein quantitative trait locus; ROSMAP, Religious Orders Study and Rush Memory and Aging Project; PWAS, proteome-wide association study

In the current work, we employed proteome-wide association studies (PWAS), a robust technique for investigating the relationship between protein abundance and disease phenotype by combining GWAS and protein quantitative trait loci (pQTL) data [15]. To validate our risk genes from multiple levels, we took a four-step approach to link the proteome and ALS. First, we integrated the large ALS GWAS data [16] and two independent human brain pQTL datasets to conduct a two-stage PWAS. The discovery pQTL data came from the ROSMAP dataset [17, 18], and the confirmatory data came from the Banner dataset [17]. We used 376 and 152 human brain proteomes, which have been the largest available human brain pQTL dataset so far. Next, to further obtain the causal relationship between candidate genes and the disease phenotype, we then applied Mendelian randomization and Bayesian colocalization analysis [19]. Finally, we verified the impact of the dysregulation of susceptibility genes found in human brain on ALS pathogenesis in various tissues using differential expression analysis of lower motor neurons, skeletal muscle, and whole blood. Figure 1 shows the detailed procedures of various analysis stages in the study.

## Materials and Methods

### ALS GWAS Data

The main analysis utilized the most recent and largest ALS genome-wide association study by van Rheenen et al. [16]. Briefly, this is a cross-ancestry GWAS meta-analysis. This analysis included 29,612 ALS patients and 122,656 control individuals and consisted of 117 independent cohorts in European ancestries (27,205 ALS patients and 110,881 control individuals) [20, 21] and the summary GWAS statistics of ALS in Asian ancestries (2407 ALS patients and 11,775 control individuals) [22, 23]. All detailed information (e.g., studied subjects, genotyping, quality control, and statistical analyses) and data availability statements can be found in the original article [16].

### Human Brain pQTL Data for Discovery PWAS

We obtained the protein abundance, referred to as protein weights, in the human brain from Wingo et al. [17], who estimated the effects of genetic variants on protein weights and generated SNP-protein abundance weights (i.e., pQTL). In addition, 376 subjects with both proteomic and genetic data were included in their PWAS. These patients exhibited different features in clinical diagnosis (including but not limited to dementia, stroke, and depression), cognitive performance, and neuropathologic traits. Furthermore, the proteomic reference dataset went through quality control procedures to identify and control the effects of clinical covariates (i.e., age, sex, and final clinical diagnosis of cognitive status) before estimating protein weights. Please refer to the original papers [17, 18] for further detailed information about proteomic profiling, protein quantification, and quality control.

### Human Brain pQTL Data for Confirmation PWAS

The other human brain pQTL data were generated from 152 subjects with both proteomic and genetic data. The human brain protein abundance included 8168 proteins after quality control. All the samples came from the dlPFC of European participants. It also passed the quality control procedures to remove the effects of clinical covariates. More detailed information can be found in the original papers [17, 24].

### Proteome-Wide Association Studies

In the discovery phase, we performed the PWAS by integrating ALS GWAS results with human brain proteomes profiled from the dlPFC (ROSMAP dataset [17]) using the FUSION pipeline. Confirmatory PWAS was performed using the same ALS GWAS and an independent set of 152 human brain proteomes profiled from the dlPFC (Banner dataset [17]). The PWAS was performed using FUSION [25] software with default settings.

### Mendelian Randomization Analysis

We further used MR analysis to validate the causal association between the identified risk genes and ALS. The brain protein abundance from the ROSMAP dataset was selected as the exposure. The SNPs included in the study robustly and independently (*R*^2^ < 0.001) predicted exposures at a genome-wide level (5 × 10^−8^). The Wald ratio calculated the log odds change in ALS risk per standard deviation change in protein biomarker in relation to the instrumenting SNP’s risk allele. MR analysis was performed using the “TwoSampleMR” package in R 4.1.02 (https://github.com/MRCIEU/TwoSampleMR).

### Colocalization Analysis

We investigated colocalization using a Bayesian colocalization method, COLOC. Colocalization was performed using the “coloc” R package [26] (http://cran.r-project.org/web/packages/coloc) and FUSION software [25]. The posterior probabilities for a shared causal variant between a pQTL and ALS significant genes in the discovery PWAS were calculated, and a high PP4 probability indicates that there is a single variant that affects both the protein expression and ALS. To confirm that the risk proteins discovered in PWAS share a common causal variant with ALS, the evidence was defined as a posterior probability of hypothesis 4 (PPH4) of 0.8 or greater [26].

### Expression Analysis of Significant PWAS Genes

To further verify the dysregulation of the significant PWAS genes in other ALS-related tissues, we collected gene expression datasets from lower motor neurons [27], skeletal muscle [28], and whole blood [29]. The original studies carried out strict quality control of the sample sources, and the expression levels of genes were normalized during the analysis process. In this study, differential expression analysis was performed in R (http://cran.r-project.org) using a two-sample *t* test. A *p* value < 0.05 was considered statistically significant.

### Gene Expression Analysis from Lower Motor Neurons

The lower motor neuron dataset was obtained from Highley et al. [27]. In their study, RNA was extracted from lower motor neurons that were isolated from the anterior horns of the postmortem cervical spinal cord. In total, there were 6 sporadic ALS patients with a mean age of 60.2 years and 6 healthy control individuals with a mean age of 61.7 years. Samples were hybridized to GeneChip Human Exon 1.0 ST Arrays, and the Partek Genomics Suite was used for normalization [27].

### Gene Expression Analysis from Skeletal Muscle

Bakay et al. [28] studied 125 human muscle biopsies from 13 diagnostic groups, including ALS, fascioscapulohumeral muscular dystrophy, Becker muscular dystrophy, and so on. Affymetrix Human Genome U133A and U133B Array were used for this gene expression analysis [28]. Here, the gene expression profile, including 9 ALS patients and 18 control individuals, and the annotation platform GPL97 were used in our analysis. Characteristics of the patient and normal volunteer samples used for mRNA profiling can be found in the original article [28].

### Gene Expression Analysis in Whole Blood

Wouter et al. [29] conducted a two-stage transcriptome-wide study, and with the corresponding whole blood gene expression profile, including 397 Netherlands ALS patients and 645 control subjects, they identified 2943 differentially expressed transcripts. The quality of isolated RNA was assessed using the Agilent 2100 Bioanalyzer system, and samples were hybridized to Illumina’s HumanHT-12 version 3 and version 4 BeadChips according to the manufacturer’s protocol (Illumina, Inc., San Diego, CA, USA) [29]. All the probes were aligned to the NCBI reference genome build 36, and expression heterogeneity was eliminated by applying surrogate variable analysis (SVA) [29]. The last dataset we used in differential expression analysis included 233 ALS patients and 508 matched control individuals.

## Results

### PWAS Identified 6 Candidate Genes Associated with ALS Using Human Brain pQTL

In the discovery phase, we performed a PWAS by integrating ALS GWAS and ROSMAP pQTL data. As a result, 6 genes (*SCFD1*, *SARM1*, *TMEM175*, *BCS1L*, *WIPI2*, and *DHRS11*) whose brain protein levels were associated with ALS were identified. Next, to validate the results, we conducted a replication study using the Banner dataset, and 2 (*SARM1* and *BCS1L*) of these 6 proteins were replicated (as shown in Table 1).

**Table 1:** Proteome-wide significant genes for amyotrophic lateral sclerosis in two datasets

		ROSMAP dataset			Banner dataset			
Gene	CHR	PWAS.*z*	PWAS.*p*	FDR.*p*	PWAS.*z*	PWAS.*p*	FDR.*p*	Replicated
*SCFD1*	14	7.32	2.56E-13	3.68E-10	-	-	-	-
*SARM1*	17	− 5.20	2.00E-07	1.44E-04	− 5.20	2.00E-07	2.24E-04	Yes
*TMEM175*	4	− 4.75	1.99E-06	9.55E-04	-	-	-	-
*BCS1L*	2	− 4.68	2.83E-06	1.02E-03	− 3.89	1.02E-04	4.86E-02	Yes
*WIPI2*	7	− 4.19	2.84E-05	8.17E-03	-	-	-	-
*DHRS11*	17	− 3.97	7.28E-05	1.75E-02	-	-	-	-

This table shows the 6 significant genes identified in the ALS PWAS and the *z* scores with their corresponding *p* values and FDR-adjusted *p* values. *CHR*, chromosome; *PWAS*, proteome-wide association study; *ROSMAP*, Religious Orders Study and Rush Memory and Aging Project

### MR Verified 3 Genes Associated with ALS

Using the ROSMAP dataset, MR analysis of brain pQTL and ALS GWAS verified that *SARM1* (OR = 0.33, *p* = 2.10 × 10^−7^), *SCFD1* (OR = 4.54, *p* = 3.72 × 10^−13^), and *DHRS11* (OR = 0.61, *p* = 7.40 × 10^−5^) provided evidence of a causal association with ALS.

### Colocalization Between ALS Risk Genes and pQTL in Human Brain

To further explore the probability that there are the same shared causal variants driving the ALS GWAS and pQTL signals, we performed colocalization analysis. The ROSMAP dataset and Banner dataset were separately used in the analysis. Two (*SCFD1* and *SARM1*) of six genes provided evidence based on PPH4 > 80% in the ROSMAP dataset, and only one gene (*SARM1*) passed replication analysis in the Banner dataset (as shown in Table 2). In particular, *SARM1* showed the most causal association with ALS in two datasets (ROSMAP PP4 = 0.999, Banner PP4 = 0.999), which indicates the potential role *SARM1* plays in ALS risk.

**Table 2:** Bayesian colocalization analysis of the 6 significant genes in the discovery ALS PWAS

Dataset	Gene	CHR	PP0^a^	PP1^b^	PP2^c^	PP3^d^	PP4^e^
ROSMAP	*SCFD1*	14	0	0	0	0.018	0.982
	*SARM1*	17	0	0.001	0	0	0.999
	*TMEM175*	4	0.240	0.044	0.055	0.009	0.652
	*BCS1L*	2	0.579	0.060	0.125	0.013	0.225
	*WIPI2*	7	0.950	0.024	0.010	0	0.016
	*DHRS11*	17	0	0.105	0	0.428	0.467
Banner	*SARM1*	17	0	0.001	0	0	0.999
	*BCS1L*	2	0.867	0.006	0.110	0.001	0.016

This table provides the results of Bayesian colocalization analysis for 6 genes identified by PWAS. Bayesian colocalization was separately conducted in the ROSMAP and Banner datasets. Based on PP4 > 0.8, only *SARM1* overlapped in the two datasets. ^a^No association with GWAS trait and pQTL trait. ^b, c^Association with either GWAS traits or pQTL traits. ^d^Association with both GWAS traits and pQTL traits with different SNPs. ^e^Association with both GWAS traits and pQTL traits with the same SNP. *ALS*, amyotrophic lateral sclerosis; *PWAS*, proteome-wide association study; *CHR*, chromosome; *ROSMAP*, Religious Orders Study and Rush Memory and Aging Project

### Expression Analysis of PWAS Genes in Lower Motor Neurons, Skeletal Muscle, and Whole Blood

**Fig. 2:**
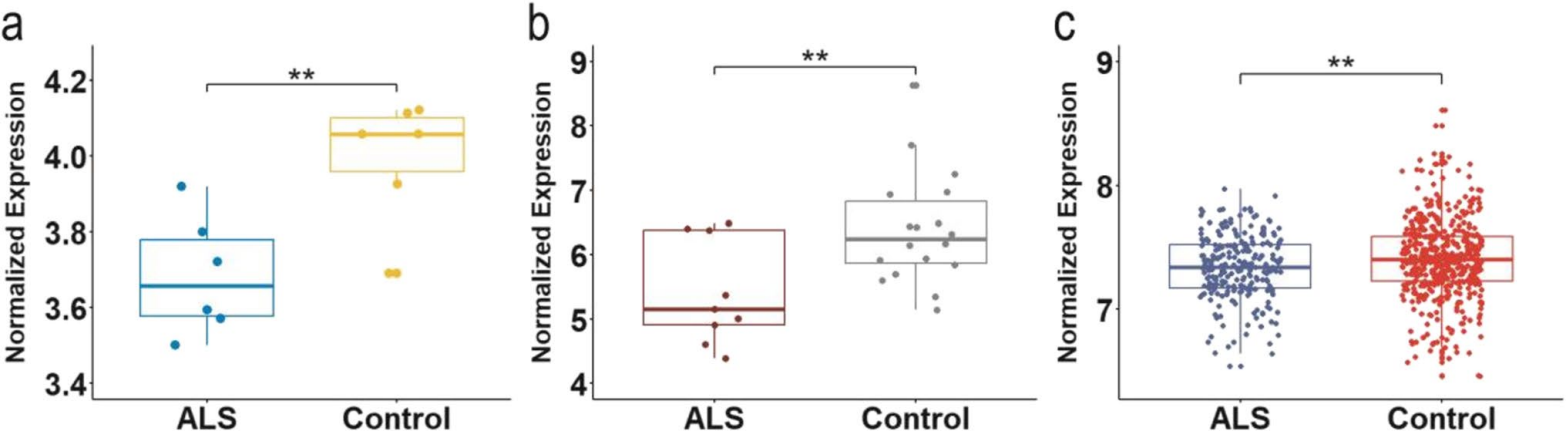
Differential expression analysis for *SARM1* validated dysregulation of risk genes at transcription level in multiple tissues. The boxplot shows the differential expression analysis results of *SARM1* between ALS patients and healthy control individuals in lower motor neurons, skeletal muscle, and whole blood. The *p* value was obtained from the *t* test. **a** N_ALS_ = 6 and N_Control_ = 6. *SARM1* was downregulated in lower motor neurons (*p* = 7.64 × 10^−3^). **b** N_ALS_ = 9 and N_Control_ = 18. *SARM1* was downregulated in skeletal muscle (*p* = 9.34 × 10^−3^). **c** N_ALS_ = 233 and N_Control_ = 508. *SARM1* was downregulated in whole blood (*p* = 1.94 × 10^−3^). ALS, amyotrophic lateral sclerosis. ^#^*p* > 0.05, *p < 0.05, **p < 0.01, ***p < 0.001

We conducted differential expression analysis using 3 datasets, and the tissue sources where RNA was extracted separately came from lower motor neurons, skeletal muscle, and whole blood. According to the *t* test, *SARM1* was downregulated in ALS patients compared with control individuals and matched the directionality of PWAS associations in lower motor neurons, skeletal muscle, and whole blood (as shown in Fig. 2). In addition, *TMEM175* (*p* = 6.42 × 10^−12^), *BCS1L* (*p* = 2.02 × 10^−7^), and *DHRS11* (*p* = 2.60 × 10^−10^) were also downregulated in whole blood with the same directionality in PWAS. Table 3 displays the consolidated results for all six genes.

**Table 3:** Results of PWAS, Mendelian randomization, Bayesian colocalization, and differential expression analysis of the six genes

Gene	PWAS	Mendelian randomization	Colocalization analysis	Differential expression analysis		
				Lower motor neurons	Skeletal muscle	Whole blood
*SARM1*	Yes	Yes	Yes	Yes	Yes	Yes
*SCFD1*	Yes	Yes	Yes	No	No	No
*BCS1L*	Yes	No	No	No	No	Yes
*TMEM175*	Yes	No	No	No	No	Yes
*DHRS11*	Yes	Yes	No	No	No	Yes
*WIPI2*	Yes	No	No	No	No	No

This table summarizes the results of the PWAS, Mendelian randomization, Bayesian colocalization, and differential expression analysis. *PWAS*, proteome-wide association study

## Discussion

In the present work, we used a two-stage approach of PWAS, MR, Bayesian colocalization, and differential expression analysis to thoroughly identify ALS risk genes. First, we identified 6 high-risk proteins (*SCFD1*, *SARM1*, *TMEM175*, *BCS1L*, *WIPI2*, and *DHRS11*) linked to ALS in the human brain. In two distinct PWAS, *SARM1* and *BCS1L* were shown to be significant. Second, we verified the causal association of *SARM1* with ALS via their cis-regulated brain protein abundance in MR analysis. Colocalization analysis also corroborated the clear causal relationship between *SARM1* and ALS. Finally, we confirmed the dysregulation of *SARM1* at the transcriptome level in three distinct tissues (lower motor neurons, skeletal muscle, and whole blood) with the same directionality as in PWAS. In addition, *BCS1L*, *TMEM175*, and *DHRS11* were verified in whole blood. Future research on ALS pathophysiology and treatment strategies could focus on these genes, particularly *SARM1*.

In the current work, we primarily focused on the ALS risk gene *SARM1* [21, 30]. *SARM1*, Sterile Alpha and TIR Motif Containing 1, encodes a protein with critical NADase activity and has been shown to be a key mediator of axon degeneration in early animal experiments [31]. Axon degeneration can be caused by activated *SARM1* due to the depletion of the axon survival factor NMNAT2 induced by injury or disease [32]. At present, preclinical studies of *SARM1* inhibitors and gene therapies targeting *SARM1* for neurodegenerative diseases are still being conducted [33, 34]. Recently, literature regarding the ALS PWAS-significant genes also indicated the abnormal expression of *SARM1* in ALS [35]. We obtained the similar results utilizing a different comprehensive analytical framework, showing that the *SARM1* risk gene has high confidence in populations of different origins. In addition, we further emphasized the tissue specificity of *SARM1* and verified its downregulation in lower motor neurons, skeletal muscle, and whole blood. Similarly, it has been shown that the loss of *SARM1* cannot slow ALS disease progression or improve ALS-associated axon degeneration in the *SOD1* mouse model of ALS [36–38]. In addition, in the mutant *TDP-43* mouse model of ALS, deleting *SARM1* can significantly attenuate motor axon and motor neuron cell body degeneration [38, 39], which indicates that the downregulation of *SARM1* could be a result of the body’s compensatory mechanisms to protect neurons from cell death. In addition to the brain and motor neurons, our study first reported the dysregulation of *SARM1* in skeletal muscle and whole blood. The downregulation of *SARM1* may affect energy metabolism by inhibiting the clearance of abnormal mitochondria in skeletal muscle and promoting the clinical manifestation of muscle weakness in ALS patients. Maintenance of appropriate NAD^ +^ levels is known to be important for mitochondrial function [40]. Additionally, *SARM1*, encoding a protein with critical NADase activity, may impair mitochondrial function by affecting the level of NAD^+^ in skeletal muscle, causing muscle damage in ALS patients. Furthermore, the dysregulation of *SARM1* in the peripheral circulation of ALS patients provided new evidence for its efficacy as a biomarker for disease monitoring. Our results suggested that *SARM1* was dysregulated in a number of ALS core affected tissues and that it may be involved in a variety of pathophysiological events contributing to the development of ALS. These results at transcription level validated our PWAS findings, which prioritized proteins that were stably differentially expressed in multiple peripheral tissues, with important implications for the identification of future drug targets. Therefore, even in its early stages, *SARM1* is very promising and has important ramifications for future studies to determine the function of *SARM1* in the onset of ALS.

The present study has several strengths. First, we used two datasets independently in each PWAS and colocalization analysis, which not only verified the preliminary outcomes but also shed light on the causal relationship through the latter analysis. The alteration of protein abundance in ALS patients’ brains implicated the involvement of novel proteins in pathological pathways as functional molecules, suggesting the effectiveness of PWAS. Second, we validated the risk genes in multiple tissues, which enhanced the reliability of our results. This provided several available tissues to identify future biomarkers and emphasized the tissue specificity of gene expression in ALS.

This study has several limitations. First, the relatively small sample size limited the output of protein abundance; thus, the pQTL data size in PWAS was limited, which explained the modest number of important PWAS genes revealed. Second, the sample size varied among the different datasets utilized in the differential expression analysis. Third, tissue sources were restricted, which may lead to a one-sided view of disease mechanisms, with the causes manifesting in multiple dimensions. (1) As a neurodegenerative illness, ALS affects the entire brain network, while the brain tissue used in PWAS only included the dlPFC. (2) We only used three peripheral tissues in the differential expression study, and other helpful tissues, such as higher motor neurons, should be investigated further. Finally, future exploration of the pathogenesis of ALS in *SARM1* cell models and animal studies is still needed.

## Conclusion

At the human brain proteome level, we discovered six ALS risk genes (*SCFD1*, *SARM1*, *TMEM175*, *BCS1L*, *WIPI2*, and *DHRS11*). Notably, *SARM1* was the most promising biomarker for ALS and was verified in multiple ALS-related tissues (lower motor neurons, skeletal muscle, and whole blood). *SARM1*-mediated axon degeneration may serve as a therapeutically targeted pathway for ALS. However, more studies are needed to confirm our results in the future.

## Data Availability

All datasets analyzed during this study are included in those published articles [16–18, 24, 27–29].
